# Growth arrest–specific 2 protein family: Structure and function

**DOI:** 10.1111/cpr.12934

**Published:** 2020-10-25

**Authors:** Nan Zhang, Chunyan Zhao, Xinxin Zhang, Xiaoteng Cui, Yan Zhao, Jie Yang, Xingjie Gao

**Affiliations:** ^1^ Department of Biochemistry and Molecular Biology Department of Immunology School of Basic Medical Sciences Tianjin Medical University Tianjin China; ^2^ Key Laboratory of Immune Microenvironment and Disease Ministry of Education Key Laboratory of Cellular and Molecular Immunology in Tianjin Excellent Talent Project Tianjin Medical University Tianjin China; ^3^ Laboratory of Neuro‐Oncology Tianjin Neurological Institute Department of Neurosurgery Tianjin Medical University General Hospital and Key Laboratory of Neurotrauma, Variation, and Regeneration Ministry of Education and Tianjin Municipal Government Tianjin China

**Keywords:** cell cycle, cytoskeleton, GAS2, GAS2L1, GAS2L2, GAS2L3

## Abstract

Members of the growth arrest–specific 2 (GAS2) protein family consist of a putative actin‐binding (CH) domain and a microtubule‐binding (GAR) domain and are considered miniversions of spectraplakins. There are four members in the GAS2 family, viz. GAS2, GAS2L1, GAS2L2 and GAS2L3. Although GAS2 is defined as a family of growth arrest–specific proteins, the significant differences in the expression patterns, interaction characteristics and biological issues or diseases among the different GAS2 family members have not been systemically reviewed to date. Therefore, we summarized the available evidence on the structures and functions of GAS2 family members. This review facilitates a comprehensive molecular understanding of the involvement of the GAS2 family members in an array of biological processes, including cytoskeleton reorganization, cell cycle, apoptosis and cancer development.

## Graphical abstract

The GAS2 (growth arrest–specific 2) family contains a putative actin‐binding (CH) domain and a microtubule‐binding (GAR) domain There are four members in the GAS2 protein family, namely GAS2, GAS2L1, GAS2L2 and GAS2L3. Different structural characteristics of GAS2 family members lead to the distinct ways for the binding with cytoskeletal proteins, and the modulation of crosslinking between actin and microtubule, which may be the mechanistic basis of versatile role of GAS2 family in cell cycle, apoptosis or cancer.

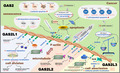

## 1 INTRODUCTION

It is widely believed that the cytoskeletal system of the mammalian cell, a type of protein fibre grid system, contains microtubules, actin and intermediate filaments, and contributes to the morphological integrity and cell polarity.^1^, ^2^ In the past decade, scientists have performed extensive studies on the biological structures and behavioural modulation mechanisms of cytoskeletal components in various cellular environments. Emerging evidence supports the finding that the dynamic co‐ordinated action between microtubules and actin filaments is closely linked to a variety of biological processes within a eukaryotic cell (eg, cell cycle and cellular migration).^1^, ^2^

Spectraplakins are a versatile cytoskeletal protein family that can crosslink the actin filaments and microtubules through two main functional domains, viz. the calponin‐homology (CH) and the growth arrest–specific related (GAR) domains.^3^ The CH domain mediates the binding ability with actin filaments, while the GAR domain participates in the interaction with the microtubules.^3^ The growth arrest–specific 2 (GAS2) family, having a relatively low molecular weight, consists of putative CH and GAR domains, and is considered a miniversion of spectraplakins.^4^ Although relevant publications regarding the structure and function of the GAS2 family members are available, this topic lacks a systemic review. For the first time, we summarized the research advances on the detailed structural characteristics and biological functions of the GAS2 family members.

## 2 STRUCTURAL INSIGHT

### 2.1 Basic structure

Four members have been identified in the GAS2 protein family, viz. GAS2, GAS2L1 (GAS2‐like protein 1), GAS2L2 (GAS2‐like protein 2) and GAS2L3 (GAS2‐like protein 3).^4^, ^5^ GAS2 protein, the first member of the GAS2 family, contains 313 amino acids (aa) and was originally defined as a ‘growth arrest–specific protein’.^6^, ^7^ In 1988, Schneider, C., et al first reported upregulated *GAS2* specific for NIH 3T3 cells in the growth‐arrest state upon the withdrawal of serum and density‐dependent inhibition.^6^ Then, in 1992 ^7^ and 1994, ^8^ Brancolini, C., et al had further produced the first polyclonal anti‐GAS2 antibody to detect a band with a molecular mass of 36 kDa using Western blotting and reported the co‐localization between GAS2 and the microfilament network system of NIH 3T3 cells for the first time.

In 1996, *GAS2L1*, also called GAS2‐related protein on chromosome 22 (*GAR22*), was identified as a tumour suppressor gene within the chromosome 22q12 region.^9^ There are two alternatively spliced mRNA transcripts of *GAS2L1*, including a shorter transcript encoding the GAS2L1α protein (337 aa), and a longer transcript encoding the GAS2L1β protein (681 aa).^10^ Analogously, *GAS2L2*, also called GAS2‐related protein on chromosome 17 (*GAR17*), encodes two kinds of protein isoforms, viz. GAS2L2α (213 aa) and GAS2L2β (880 aa).^10^ Human *GAS2L3*, the last identified member of the GAS2 family, is situated on chromosome 12 and encodes 694 aa with a protein molecular weight of approximately 75.2 kDa.^4^

**Figure 1:**
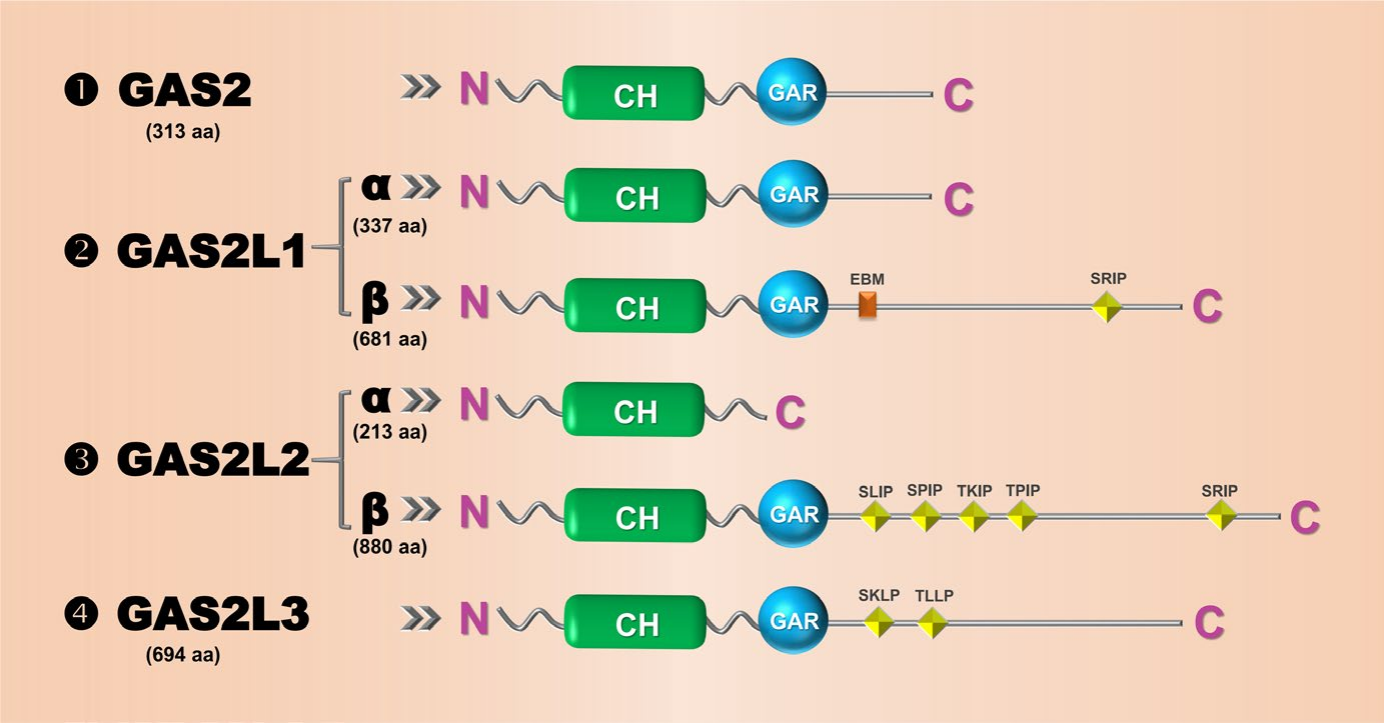
Schematic view of the structure of the GAS2 protein family members. The information of GAS2, GAS2L1α, GAS2L1β, GAS2L2α, GAS2L2β and GAS2L3 is shown, respectively. CH, calponin‐homology; GAR, growth arrest–specific related; EBM, EB‐binding motif; SRIP, SLIP, SPIP, TKIP, TPIP, SKLP and TLLP are the conserved motifs

**Figure 2:**
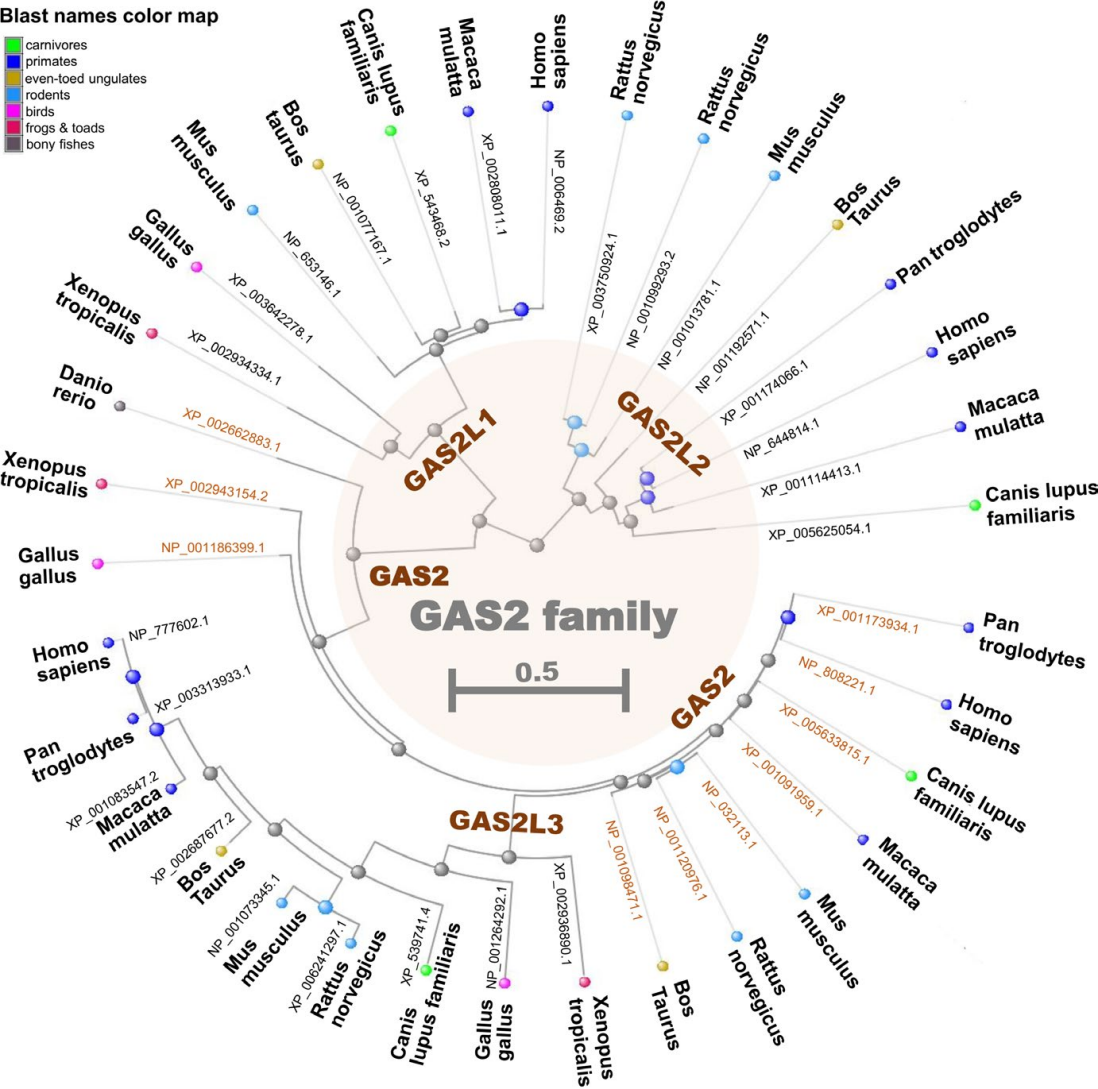
Phylogenetic tree of the GAS2 family members. Using the constraint‐based multiple alignment tool (COBALT) of National Center for Biotechnology Information (NCBI) [https://www.ncbi.nlm.nih.gov/tools/cobalt/], the gene tree of GAS2, GAS2L1, GAS2L2 and GAS2L3 among different species is established. The respective protein accessions of NCBI are provided

As stated above, the N‐terminal CH domain that binds the actin, followed by the GAR domain that binds the microtubule are the two essential elements of the protein structure within the GAS2 family. Reports reveal that different GAS2 family proteins exhibit certain structural characteristics.^4^, ^5^, ^10^, ^11^ As shown in Figure 1, the GAS2, GAS2L1α, GAS2L1β, GAS2L2β and GAS2L3 proteins contain both CH and GAR domains, whereas GAS2L2α contains only the CH domain. The Ser‐x‐Ile‐Pro (SxIP, x represents any aa) motif‐containing microtubule positioning signals (MtLSs) are present at the C‐terminus of GAS2L1β, GAS2L2β and GAS2L3 proteins. Further, the phylogenetic tree of the GAS2 family members (Figure 2) indicates the structural conservation of GAS2, GAS2L1, GAS2L2 and GAS2L3 among different species.

### 2.2 Expression pattern

**Figure 3:**
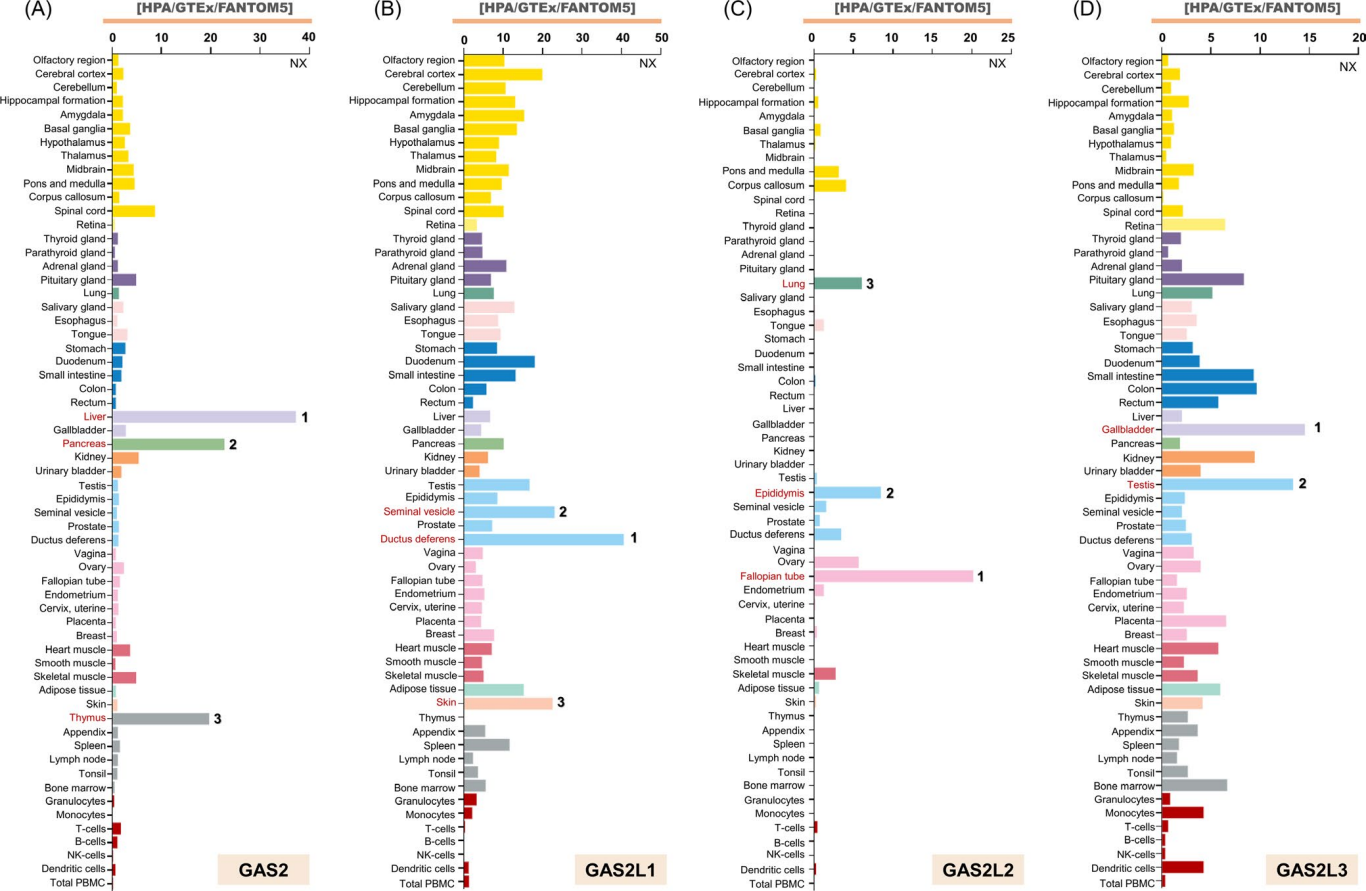
Expression profiles of the GAS2 family members in different normal tissues or blood cells. Based on the consensus datasets of Human Protein Atlas (HPA), Genotype‐Tissue Expression (GTEx) and Functional Annotation of the Mammalian Genome 5 (FANTOM5), the RNA Expression level of GAS2 (A), GAS2L1 (B), GAS2L2 (C) and GAS2L3 (D) in fifty‐five types of tissues or six types of blood cells (https://www.proteinatlas.org/search) is documented. The tissues with relatively high expression levels are marked in red. NX, consensus normalized expression level

Here, we have provided an overview of the expression of the GAS2 family members in different normal tissues or blood cells. As shown in Figure 3, *GAS2*, *GAS2L1*, *GAS2L2* and *GAS2L3* exhibit distinct distribution characteristics. *GAS2* is highly expressed in the liver, pancreas and thymus tissues, whereas *GAS2L1* is highly expressed in the tissues of ductus deferens, seminal vesicles and skin. The expression level of *GAS2L2* is high in the fallopian tube, epididymis, and lung tissues, whereas the expression level of *GAS2L3* is high in the tissues of the gallbladder and testis. In addition, several studies have investigated the potential modulation mechanisms of the GAS2 family members at the transcriptional level. For instance, stimulation of quiescent NIH 3T3 cells decreases the *GAS2* mRNA levels, which is independent of protein synthesis.^12^ GAS2 can be translationally regulated by several molecules, such as hepatitis B virus (HBV)–encoded X antigen with 35 aa deleted at the C‐terminus (HBxΔ35), ^13^ or eIF4E‐binding proteins (4E‐BPs).^14^ In addition, *GAS2L1* may be transcriptionally regulated by OCT4, SOX2 or NANOG in human embryonic stem cell lines.^15^ The mammalian DP, RB‐like, E2F and MuvB (DREAM) complex takes part in the transcriptional activation of *GAS2L3* in HeLa cell lines.^16^

### 2.3 Subcellular localization

Current evidence suggests that the GAS2 family members belong to the cytoskeletal localization proteins. In NIH 3T3 cells, GAS2 protein was found to co‐localize with stress fibre upon serum starvation.^7^ Hyperphosphorylated GAS2 protein is enriched at the membrane ruffles of the cell periphery upon the addition of 20% foetal calf serum (FCS).^8^ For the GAS2‐injected *Xenopus* embryos, co‐localization between GAS2 and the microtubules at the cell cortex is observed.^17^

Both GAS2L1α and GAS2L2α interact and localize with the actin filaments (especially the stress fibres), but not the microtubules, despite the presence of a GAR domain within GAS2L1α. Conversely, GAS2L1β and GAS2L2β bind to both microfilaments and microtubules via their CH and GAR domains, and are predominantly localized with the actin filaments, while partly localizing with the microtubules in some cells.^10^, ^18^ In addition, a potential MtLS and a non‐canonical end‐binding (EB) protein‐binding motif (EBM) for the GAS2L1β, and five MtLSs for GAS2L2β are important for binding with the EB protein and localization with the plus‐ends of microtubules.^5^, ^11^ GAS2L1β was also reported to localize at the proximal end of two mature centrioles in the G1 phase^19^ and accumulate at the mitotic structures, such as the contractile ring, cleavage furrow and mitotic spindle.^18^

Likewise, GAS2L3 worked as a type of microfilament and microtubule crosslinking protein, based on the cytoskeleton‐binding properties of the CH domain and C‐terminus.^4^ Furthermore, GAS2L3 localizes with the mitotic spindle mid‐zone of anaphase via its C‐terminus, the microtubule‐rich mid‐body of cytokinesis through the CH and GAR domains, ^16^, ^20^ and the constriction zone of abscission via the binding of the GAR domain and chromosomal passenger complex.^21^

**Figure 4:**
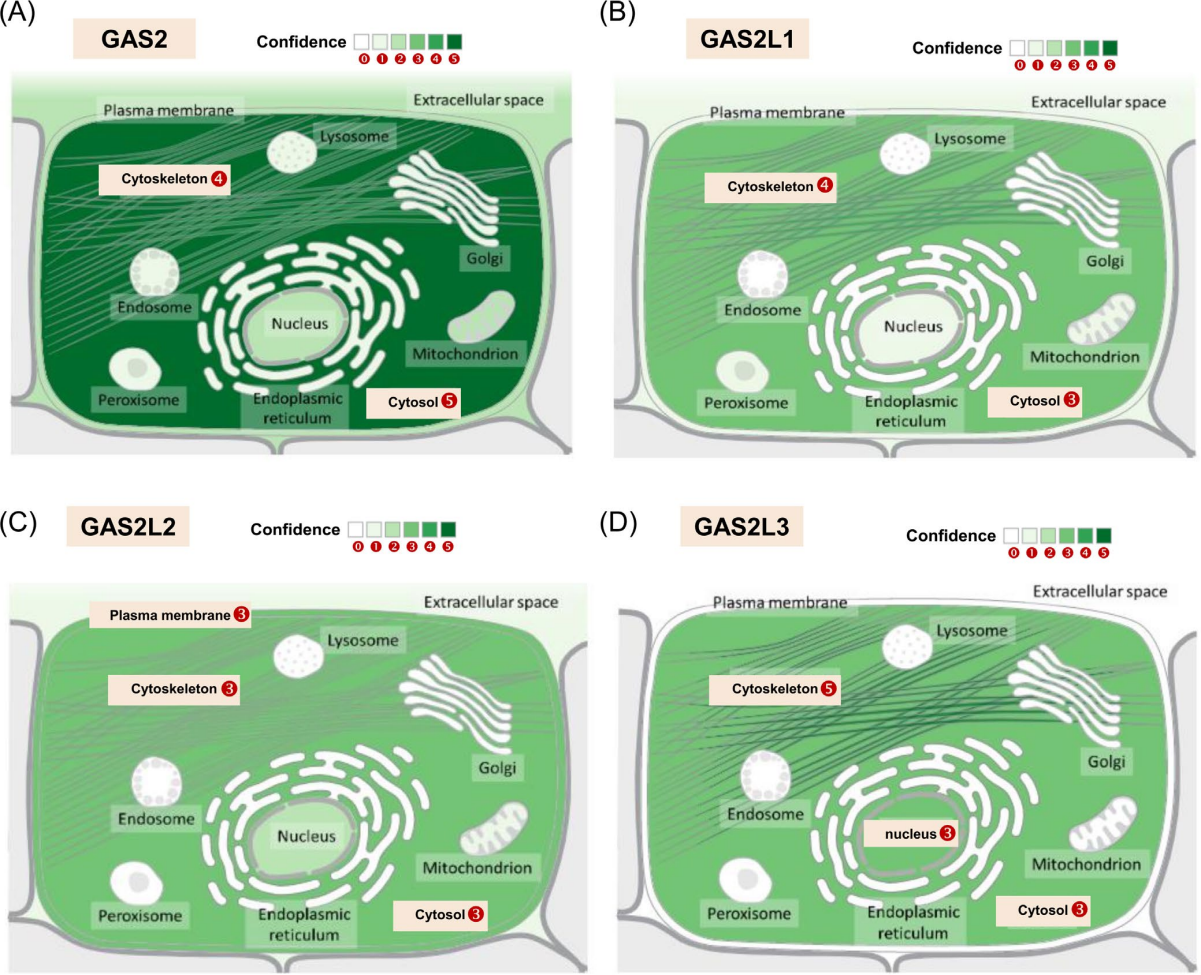
The subcellular localization of the GAS2 family members. Through the web portal of GeneCards **(** https://www.genecards.org/), COMPARTMENTS localization information of GAS2 (A), GAS2L1 (B), GAS2L2 (C) and GAS2L3 (D) is provided, respectively. The respective unified confidence scores (1 ~ 5) of the protein subcellular localization evidence are shown

Herein, we have also summarized the protein subcellular localization of the GAS2 family members, based on COMPARTMENTS localization data, which provides the unification and visualization of protein subcellular localization evidence. Indeed, there are relatively high confidence scores for the localization of the cytoskeleton and cytosol for all GAS2 family members (Figure 4). Additionally, we observed high confidence for the subcellular localization of GAS2L2 in the plasma membrane and the localization of GAS2L3 in the nucleus.

### 2.4 Phosphorylation modification

We obtained several investigations regarding the phosphorylation modification of GAS2, ^7^, ^8^ GAS2L1 ^22^, ^23^ and GAS2L3, ^20^, ^21^ but not GAS2L2, from the retrieved database. In 1992, Brancolini, C., et al demonstrated that treatment with 20% FCS can lead to the hyperphosphorylation of GAS2 protein in serum‐starved NIH‐3T3 cells.^7^ In 1994, they further found that the GAS2 protein can be phosphorylated on serine residues.^8^ However, the phosphorylation site of the GAS2 protein has not been studied in detail. In serum‐starved 3T3‐L1 adipocytes, GAS2L1 can encounter insulin‐regulated phosphorylation at Ser600 and Ser602 sites of NP_006469.2.^22^ Recently, Au, F. K. C., et al reported that the Ser352 site of GAS2L1 protein can be phosphorylated by Nek2A in the G2/M phase of the cell cycle.^23^ In addition, the Ser307 and Ser607 sites of GAS2L3 are also found to be phosphorylated by cyclin‐dependent kinase (CDK) 1 (CDK1) in late mitosis phase of HEK293 cells. ^20^ Considering the lack of reports regarding the detailed phosphorylation sites, or any other potential functional phosphorylation status of the GAS2 family members, we predicted the phosphorylation sites of GAS2, GAS2L1, GAS2L2 and GAS2L3. As shown in Figure S1, we observed that the phosphorylation of the Ser282 site within the GAS2 protein was supported by most studies with high/low‐throughput analyses (Figure S1A). For GAS2L1, apart from the reported Ser352 (S352) site, approximately fifty papers provided evidence regarding the potential phosphorylated Ser306 (S306) site (Figure S1B). However, there were no relatively potential phosphorylation sites for GAS2L2 (Figure S1C). With regard to GAS2L3, apart from the reported CDK1‐mediated Ser307 (S307) and Ser607 (S607) phosphorylation, more than twenty studies supported the potential phosphorylated Ser376 (S376) site (Figure S1D). This hints at the complexity of the phosphorylation mechanism among the GAS2 family members.

## 3 BIOLOGICAL FUNCTION

To date, emerging evidence has functionally linked the GAS2 protein family to a series of physiological and pathological processes, such as cytoskeletal regulation, cell cycle, apoptosis, senescence, differentiation and cancer. Distinct biological events co‐operate to maintain the normal physiological function of the cell; abnormal co‐operation can result in clinical pathology.

### 3.1 Cytoskeleton

Modulation of the cytoskeletal system is implicated in various biological processes of the GAS2 family and can be considered the most important functional feature of GAS2, GAS2L1, GAS2L2 and GAS2L3. Here, we summarized some evidence regarding the fundamental regulatory role of the GAS2 family members. For instance, the exogenous overexpression of the C‐terminal deleted derivatives of GAS2 in NIH 3T3 fibroblasts, but not the wild‐type GAS2 protein, can especially lead to a dramatic alteration of the cell shape and microfilament system, rather than the intermediate filament or microtubule system.^24^ Similarly, during apoptosis, the proteolytic cleavage of the C‐terminal domain of GAS2 is coupled with the specific microfilament rearrangement, but does not affect the binding of GAS2 and F‐actin.^25^

The crosslinking between actin and microtubules using cytoskeletal regulators is instrumental for various biological processes, such as cell morphology, contact, polarity, division and movement.^1^ GAS2L1β and GAS2L2β can interact with the EB protein through the SxIP or SxLP motifs to regulate microtubule dynamics, thereby mediating the efficient crosstalk between microfilaments and microtubules.^5^ Apart from the classical SxIP motifs, non‐canonical EBM at the C‐terminus mediates the binding of GAS2L1β and EB protein 1 as well.^11^ The actin‐microtubule cytolinker property of GAS2L1β is involved in the process of normal cell division.^18^, ^19^ As a regulator of cell adhesion and migration, the deficiency of GAS2L1β can impair the actin dynamics and the typical axoneme ultrastructure of spermatozoa in mice.^11^ In addition, GAS2L1β exerts an effect on the axon morphology of rat hippocampal neurones.^26^ GAS2L2β has been reported to bind an A2A adenosine receptor (A2AR) and facilitate cAMP signalling and recruitment of A2AR signalosome to the F‐actin‐containing microenvironment.^27^ A recent study by Bustamante‐Marin, XM, et al showed that conditional deletion of GAS2L2 in mice exhibited a primary ciliary dyskinesia‐like phenotype, with impaired cilia orientation and mucociliary clearance.^28^ In addition, the loss of GAS2L3 inhibits the proliferation of cardiomyocytes and impairs cardiomyocyte cytokinesis during the embryonic development of mice.^29^

### 3.2 Cell cycle

A growing number of proteins have been reported to be associated with the modulation of cell cycle progression by influencing specific cell cycle proteins, such as cyclin‐dependent kinases (CDKs).^30^, ^31^ GAS2 protein was initially identified in mammalian cells with cell cycle arrest.^6^, ^7^ Subsequently, several reports explored the distinct correlation between *GAS2* and cell cycle in different cells. For example, upon serum starvation, GAS2 protein was upregulated in the murine 3T3 cell line,^6^, ^7^ and slightly upregulated in the F9 embryonal carcinoma cells,^32^ but not in the murine keratinocyte cell line MSCP5, suggesting the cell specificity of GAS2 function.^33^ *GAS2* is differentially expressed in mouse primary hepatocytes of different ploidy.^34^ GAS2 overexpression inhibits cell division in *Xenopus* embryos and results in the presence of multinucleated cells.^17^ Similarly, upregulated GAS2 in a hepatocellular carcinoma (HCC) cell line (SK‐Hep1) suppresses the G1‐to‐S transition of the cell cycle.^13^, ^35^ In contrast, GAS2 in two T‐cell acute lymphoblastic leukaemia cell lines (Jurkat and CCRF‐CEM) facilitates the G1‐to‐S transition of the cell cycle.^36^

To date, limited experimental evidence has been found linking the GAS2 family members with the cell cycle. For example, overexpression of GAS2L1 was reported to lengthen the cell cycle and inhibit the growth of stem cell factor/erythropoietin (SCF/EPO)‐dependent red cell progenitors.^37^ Nevertheless, GAS2L1 depletion or the expression of GAS2L1 mutants without EB protein‐binding ability can also lead to aberrant cell division and multinucleation in U2OS cells.^18^ The link between GAS2L2 and cell cycle has not yet been investigated.

Unlike GAS2, *GAS2L3* mRNA expression in resting cells under serum starvation is extremely low. On the contrary, the expression level gradually increases when the cell re‐enters the cell cycle and reaches a peak in the G2/M phase.^16^ The normal expression of GAS2L3 is important for abscission in the last step of HeLa cell cytokinesis, ^16^, ^20^, ^21^, ^38^ and for cardiomyocyte cytokinesis in mice.^29^, ^39^ The cell‐free systems from both, *Xenopus laevis* egg and human HEK293 cells, were utilized by Pe'er, T., et al In 2013, a study revealed that GAS2L3 protein serves as a substrate of anaphase promoting complex/cyclosome (APC/C) and can be degraded in a Cadherin 1 (CDH1)‐mediated ubiquitin proteasome pathway.^20^ The destruction box (D‐box) with ‘^610^R/T/P/L^613^’ motif of GAS2L3 was thought to be essential for GAS2L3 degradation in G1 extracts.^20^

### 3.3 Apoptosis

As we know, apoptosis is the autonomous and orderly death of cells controlled by the expression and activation of various genes.^40^ Accumulating evidence from the past decades suggests an association between GAS2 and apoptosis tissue, whereas only one publication for GAS2L3, ^41^ and no relevant publication for GAS2L1 or GAS2L2 are available. As reported by Asai, T., et al in 2012, the overexpression of GAS2L3 protein resulted in an enhanced apoptosis level of Lin‐Sca‐1 + c‐Kit + CD48‐CD150 + cells isolated from mouse foetal liver cells and increased the radiosensitivity of haematopoietic stem cells in mice.^41^ We have provided available evidence regarding the association between GAS2 and apoptosis, as follows.

The high expression level of GAS2 protein was reportedly associated with its susceptibility to p53‐dependent apoptosis in BALB/c cell lines, with perturbed phosphorylated retinoblastoma (p‐Rb) function under low serum conditions.^42^ In the U2OS cell line, GAS2 can interact with m‐calpain, inhibit calpain activity, and enhance the stability and transcriptional activity of p53 protein, thereby increasing cellular susceptibility to apoptosis upon stimulation by DNA‐damaging agents.^43^ Similarly, GAS2 overexpression can increase the etoposide‐induced apoptosis in the MCF7 cells with wild‐type p53, but not in PC3, a type of prostate cancer cell line lacking p53.^44^ GAS2 was reported to retard the cellular growth of HCC by enhancing p53‐dependent apoptosis.^13^, ^35^ In chicks, probiotics‐induced high expression of GAS2 may be linked to the increased apoptosis of caecal cells upon *Salmonella* infection.^45^ In fish, the high expression level of *GAS2* seems to be associated with the cold stress–induced apoptosis of liver cells in tilapia (*Oreochromis niloticus*).^46^ Hence, GAS2 seems to work as a pro‐apoptotic factor.

However, we observed the opposite result in leukaemic cells. For chronic myeloid leukaemia (CML), Huang et al provided evidence that the high expression level of GAS2 is associated with increased β‐catenin‐dependent survivin expression, resulting in a Fas‐induced apoptosis resistance status in Bcr‐Abl‐positive or ICSBP‐knockout myeloid progenitor cells.^47^ Similarly, GAS2 can suppress the apoptosis status and chemotherapeutic sensitivity of Jurkat, CCRF‐CEM or K562 cells.^36^, ^48^ It is noteworthy that, in leukaemia cells, GAS2 also acts as an inhibitor of calpain activity.^47^ The different cellular downstream mechanisms of calpain, that is, p53‐dependent or β‐catenin‐dependent apoptosis, partly contribute to this inconsistency.

Notably, during apoptosis, the C‐terminus of GAS2 protein can be cleaved in apoptotic mammalian cells, and the Asp residue at the 279th site of GAS2 is essential for the caspase 3/7‐mediated apoptotic response of GAS2.^24^, ^25^, ^49^ The GAS2 proteolytic cleavage is coupled to the reorganization of microfilament architecture and the change of cell morphology during apoptosis, ^24^, ^25^, ^49^ although it fails to abrogate the binding of GAS2 and F‐actin.^25^ Further, Lee, K. K., et al reported that the involvement of GAS2 in apoptosis is related to chondrogenesis in mice.^50^ Thus, GAS2 protein probably acts as a dual regulator of cell growth and cell death, which mainly involves the mechanism of GAS2 protein expression alteration and apoptotic structure truncation.

### 3.4 Cancer

The potential roles of GAS2 protein in tumorigenesis of liver cancer,^13^, ^35^ leukaemia,^36^, ^48^, ^51^, ^52^, ^53^, ^54^ recurrent colorectal cancer,^55^, ^56^, ^57^ prostate cancer,^58^ breast cancer ^44^, ^59^ or lung adenocarcinoma ^60^ have been explored, but there are very few studies related to GAS2L1, GAS2L2 and GAS2L3. *GAS2L1* was markedly diminished in acute myeloid leukaemia,^61^ and the mutation of *GAS2L1* may be associated with the neoplastic transformation of meningioma.^62^ The rs12602590 polymorphism of *GAS2L2* was reported to be linked to the pain flare and dexamethasone response of cancer patients with painful bone metastases who received palliative radiation therapy.^63^ *GAS2L3* is downregulated in gastric cancer HSC45‐M2 cells following treatment with ^213^Bi‐d9Mab.^64^

Here, we have provided a brief overview of the research advances of the oncogenic roles of GAS2 family in liver cancer and leukaemia. *GAS2* mRNA can be detected in the majority of tissues, with the highest levels of GAS2 protein in the liver, lung and kidney tissues.^49^ Nevertheless, a lower expression level of GAS2 had been detected in clinical HCC tissues than in the corresponding normal tissues.^13^, ^35^ GAS2 can inhibit the carcinogenesis of HCC by influencing the cell cycle and p53‐dependent apoptosis.^35^ Additionally, HBxΔ35 can downregulate the expression of *GAS2* by binding to the promoter region of *GAS2*, thereby resulting in a reduced cell apoptosis level and facilitating the pathogenesis of liver cancer.^13^

Compared with control lymphocytes, GAS2 protein is upregulated in a group of leukaemic cell lines (eg, SHI‐1, Jurkat, CCRF‐CEM, or THP‐1 cells).^36^, ^51^ Similarly, there is a higher expression level of GAS2 protein in the nucleated cells from CML patients than those from the healthy donors.^48^ Moreover, we detected a high expression level of GAS2 in the acute myeloid leukaemia and lymphoid neoplasm diffuse large B‐cell lymphoma samples of The Cancer Genome Atlas (TCGA) cohorts, when compared with the normal controls of Genotype‐Tissue Expression (GTEx) database (data not shown). GAS2 protein has been reported to promote the growth of several leukaemic cells, such as K562, MEG‐01, Jurkat or THP‐1 cells.^36^, ^48^, ^51^ Further, GAS2 can facilitate the tumour formation of THP‐1 cells in nude mice.^51^ GAS2 is reportedly involved in the disease transformation of CML ^52^ and exhibits an opposite effect on the inhibition or emergency granulopoiesis termination roles of ICSBP for leukaemia.^53^, ^54^ In such a scenario, GAS2, therefore, tends to be an oncogene of leukaemia, which is different from the suppressor role of GAS2 in HCC. This is also in line with the above statement regarding the distinct roles of GAS2 expression in the cell cycle or apoptosis of leukaemic and HCC cells.

**Figure 5:**
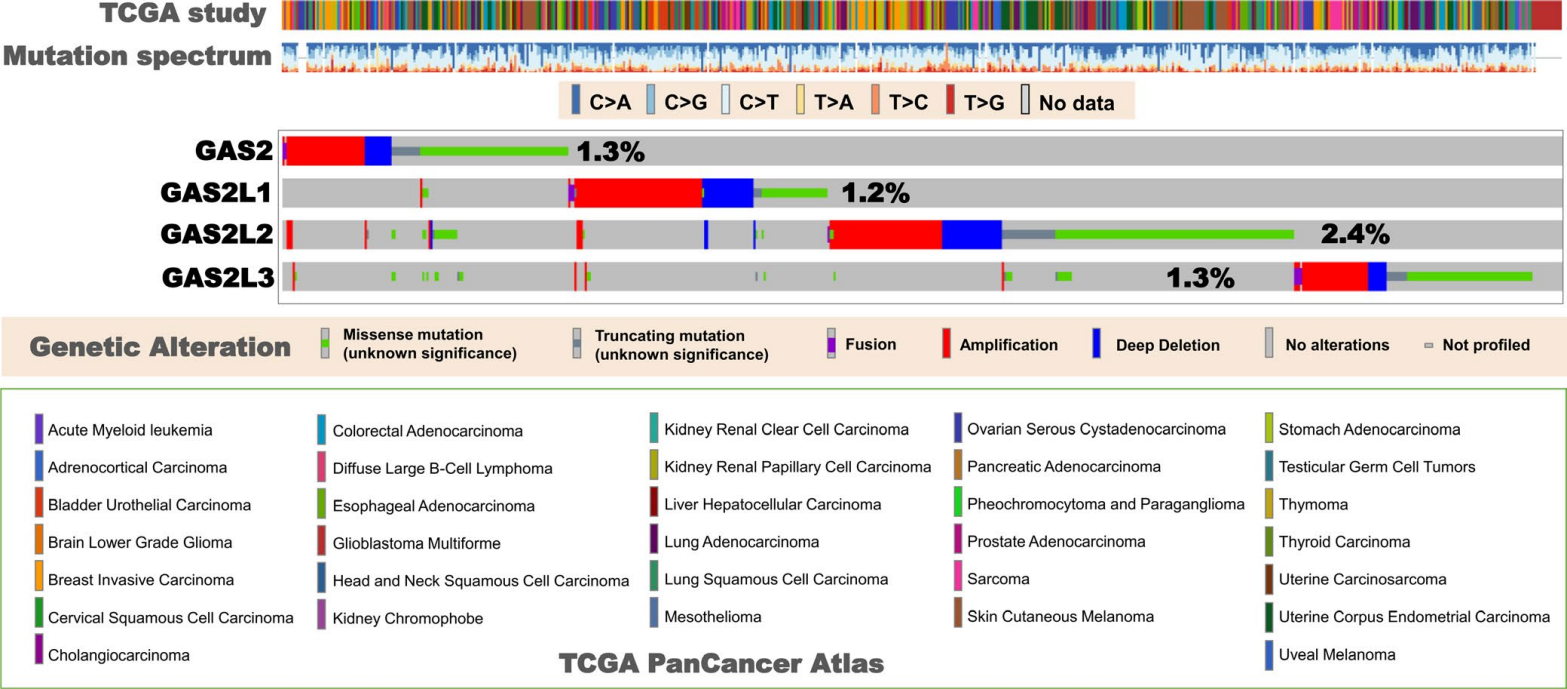
Mutational profile analysis of the GAS2 family members in TCGA tumours. The gene mutation characteristics of the GAS2 family members in different types of tumours within TCGA databases are explored using the cBioPortal online website (https://www.cbioportal.org/). The information on mutation type, spectrum and frequency is provided

To date, very little is known about the correlation between genetic alterations in the GAS2 family and tumours. Herein, we analysed the mutation characteristics of the GAS2 family members in different types of cancers, based on the data from TCGA database. Figure 5 presents the mutation rates of GAS2 (1.3%), GAS2L1 (1.2%), GAS2L2 (2.4%) and GAS2L3 (1.3%) in the whole tumour samples. Interestingly, we did not observe an obvious overlap of tumour patients with genetic mutations among the four GAS2 family members. We also analysed the mutation frequency of specific mutation sites after pooling all the cancer cases (Figure S2), but could not identify the specific mutation site with high frequency. As shown in Figure S2A, for GAS2 protein, the mis‐sense mutation of S133L/E134Kfs*17 with the highest frequency was only detected in four cancer cases. No mutation site with relatively high frequency was identified for GAS2L1 (Figure S2B) and GAS2L3 (Figure S2D). A mis‐sense mutation site (R122Q/*/L) and a truncating mutation site (P409Lfs*92) of GAS2L2 were detected in six cancer cases (Figure S2C).

### 3.5 Others

We retrieved relevant publications regarding the association between GAS2 and senescence. In murine embryonic fibroblasts (MEFs), GAS2 protein is upregulated during replicative senescence ^65^ and works as a p53‐stabilizing protein to engage in the p53‐dependent senescence.^14^ Another study showed that GAS2 protein can promote the senescence of HCC SK‐Hep1 cell lines.^13^ However, there is still a lack of potential links between other the GAS2 family members and senescence.

Several cell or animal‐based assays indicate the potential role of the GAS2 family members in the differentiation and development of cells or tissues. GAS2 was reportedly associated with oocyte cyst breakdown, follicular development of mice ^66^ and patellar tendon healing process in rats.^67^ GAS2L1 can function as the target gene of the thyroid hormone receptor to be linked to red blood cell differentiation.^37^ Compared with the non‐diabetic littermates, the protein level of GAS2L1α was significantly increased in the glomerular podocytes of diabetic mice, suggesting a potential role of GAS2L1α in the development of diabetic disease.^68^ GAS2L1β was reportedly linked to the spermatid elongation and axoneme development in mice,^11^ and the neuronal development in rats.^26^ As already indicated, GAS2L3 is closely associated with the brain morphogenesis and development in zebrafish.^69^

**Figure 6:**
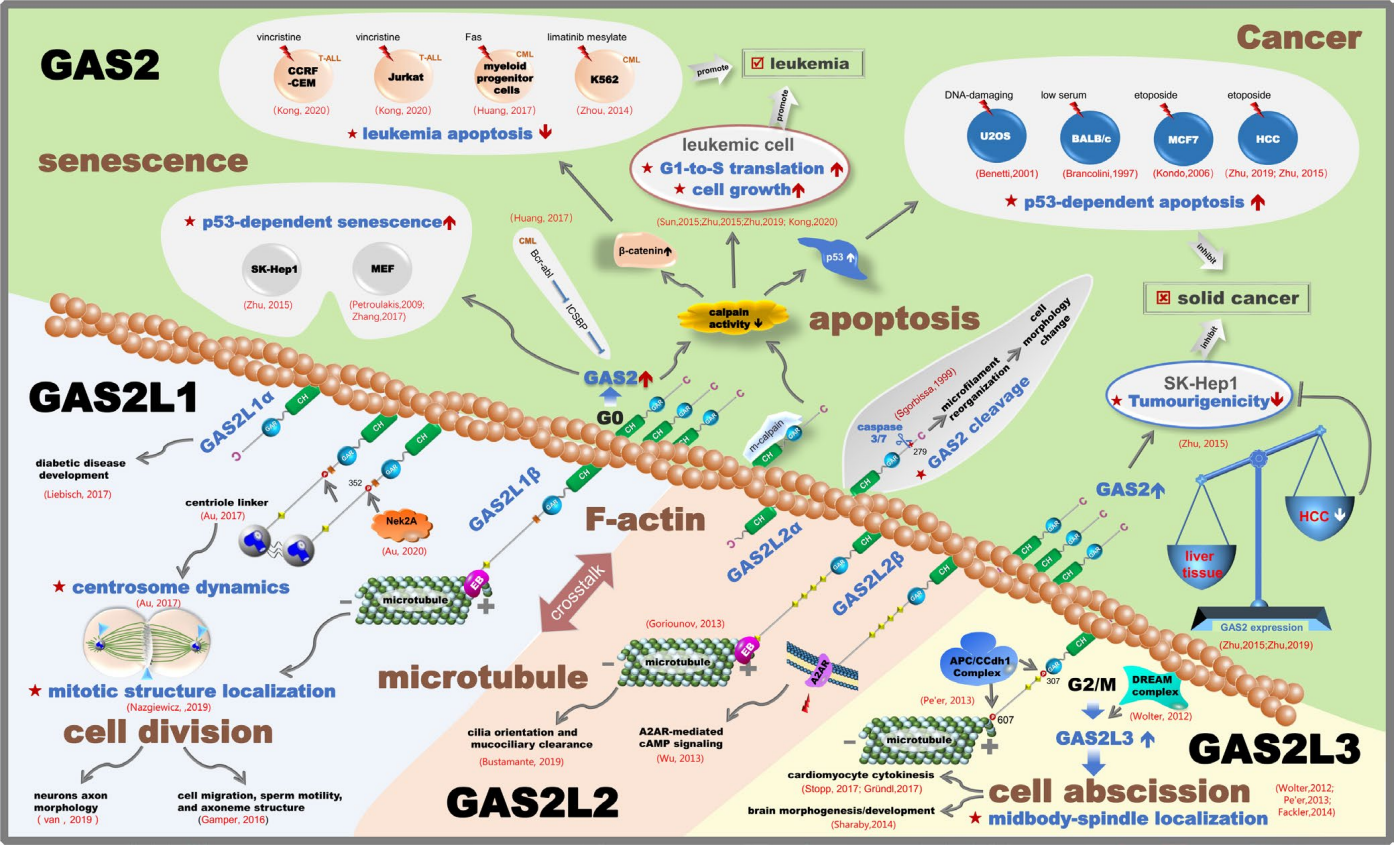
Schematic representation of the biological functions of the GAS2 family members

Based on the above statements, we have summarized the main biological functions of the GAS2 family members and provided the relevant schematic representation in Figure 6. Different structural characteristics of the GAS2 family members result in distinct pathways for binding with cytoskeletal proteins, and the regulation of crosslinking between actin and microtubules, which may be the mechanistic basis of the versatile role of the GAS2 family in cell cycle, apoptosis and cancer.

## 4 CONCLUSIONS

Even though the GAS2 family was originally defined as a family consisting of growth arrest–specific proteins, there are distinct and even opposite expression patterns or regulation mechanisms during the cell cycle for the different members. Interestingly, we observed the opposite effect of GAS2 protein on cell cycle, apoptosis and tumorigenesis issues between solid cancer (especially HCC) and leukaemia. It is yet to be understood whether the GAS2 protein displays different cytoskeletal regulatory mechanisms in solid and non‐solid tumour cells, which is responsible for the dual anti‐tumour and tumour‐promoting role. To address this, more in vivo and in vitro evidence of expression level modulation or post‐translational modification of the GAS2 family members is required. Additionally, more cell, animal and clinical sample‐based investigations are needed to further explore the potential role or clinical benefits of GAS2L1, GAS2L2 and GAS2L3 in the aetiology and pathogenesis of relevant diseases, such as tumours.

## CONFLICT OF INTEREST

The authors declare that they have no conflict of interest.

## AUTHORS CONTRIBUTIONS

JY and XG conceived the idea and designed the study; XG, NZ and CZ wrote the manuscript; NZ, CZ, XZ and YZ read and organized the literature; and XC revised the manuscript critically. All authors have read and approved the final manuscript.

## Supporting information

Fig S1

Fig S2

Supplementary Material

## Data Availability

Data available on request.
