# Supplementary figures and images for: Growth arrest–specific 2 protein family: Structure and function
Source: Cell Prolif. 2020 Oct 25;54(1):e12934. doi: 10.1111/cpr.12934 (PMC7791176; doi:10.1111/cpr.12934)

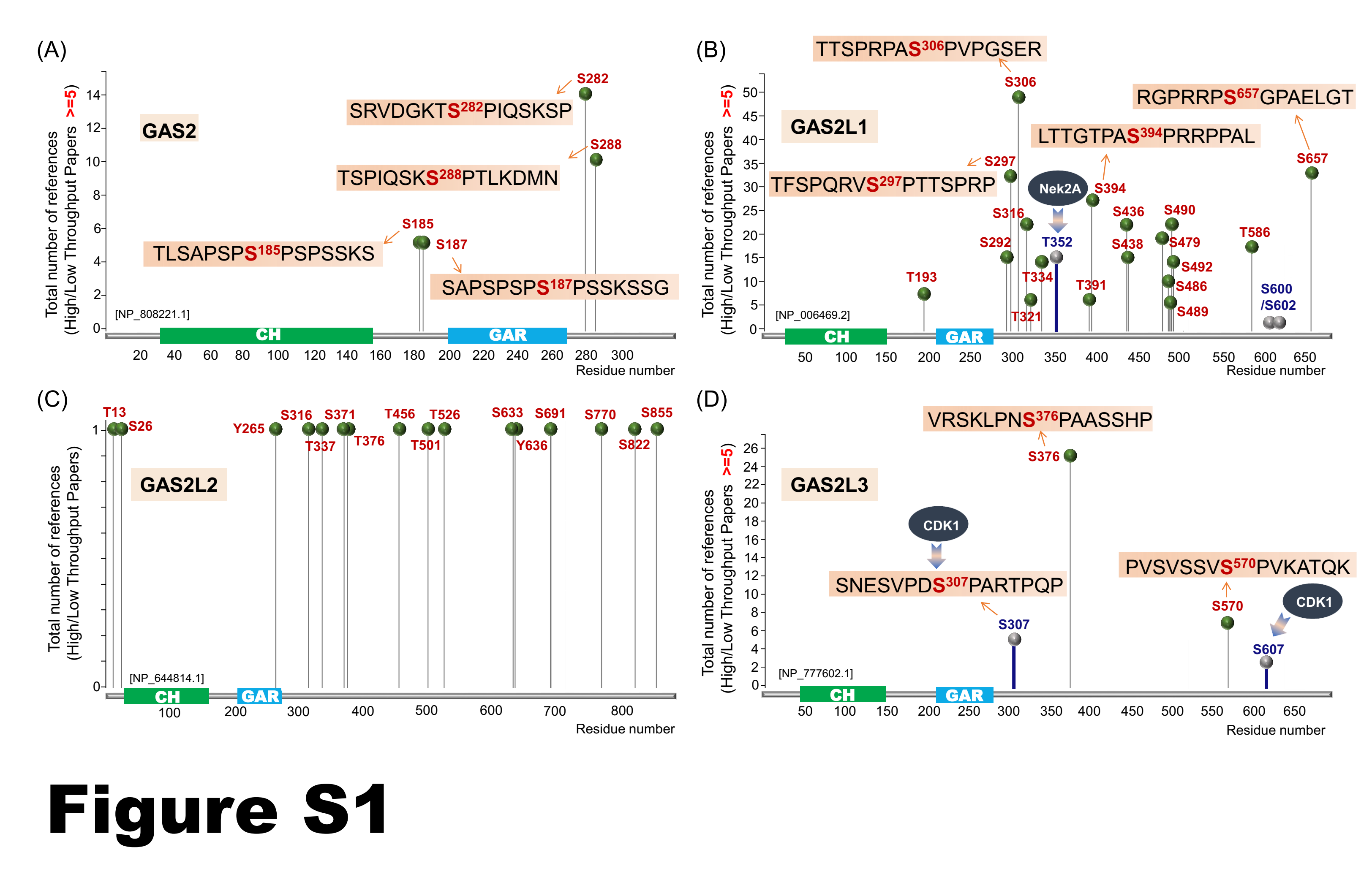

Supplement: Supplementary file 1 — Fig S1 [file CPR-54-e12934-s001.tif]

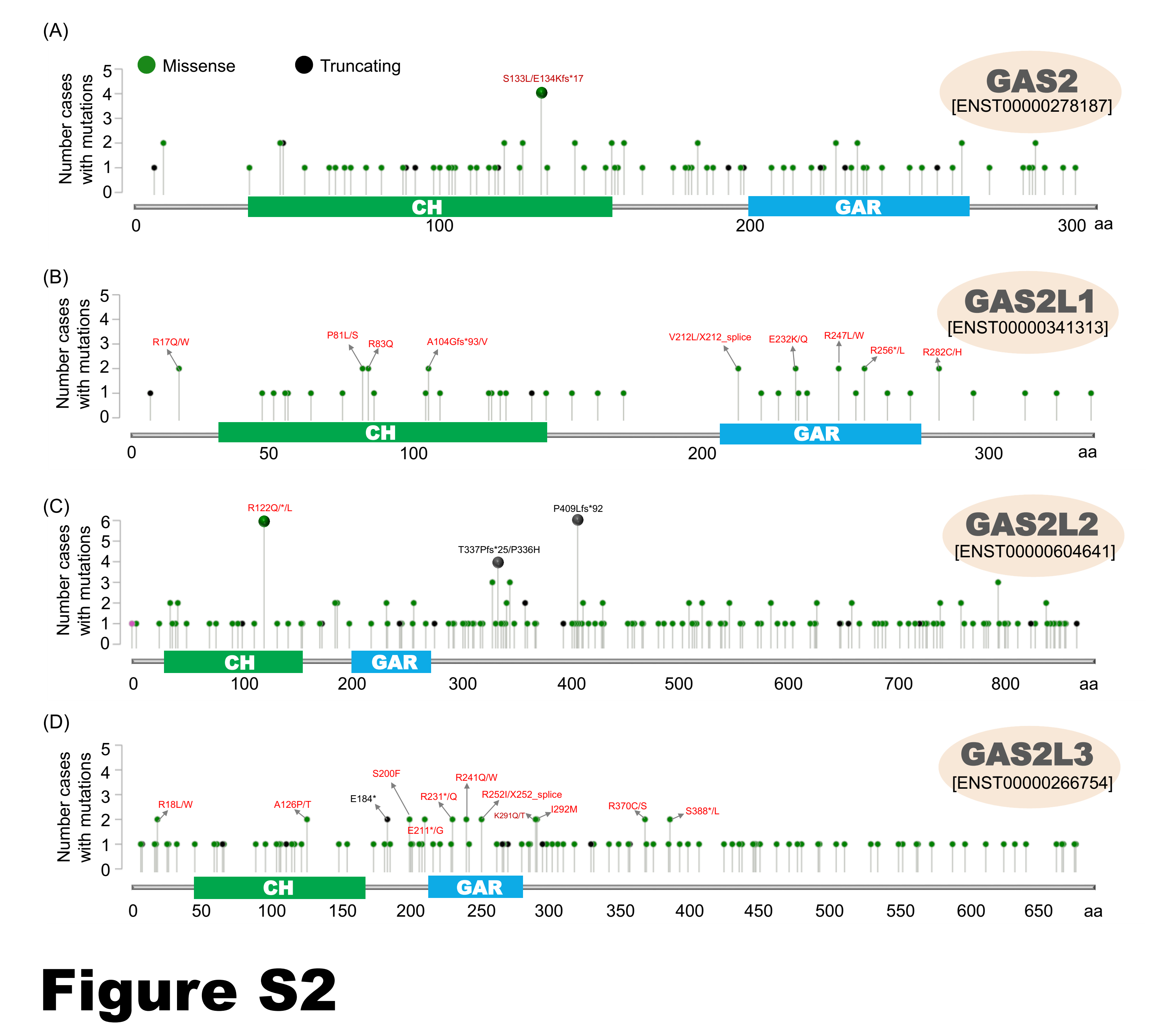

Supplement: Supplementary file 2 — Fig S2 [file CPR-54-e12934-s002.tif]
