# Supplementary material for: Growth arrest–specific 2 protein family: Structure and function
Source: Cell Prolif. 2020 Oct 25;54(1):e12934. doi: 10.1111/cpr.12934 (PMC7791176; doi:10.1111/cpr.12934)
Supplement: Supplementary file 3 — Supplementary Material [file CPR-54-e12934-s003.docx]

# SUPPLEMENTARY DATA

### FIGURE S1. Phosphorylation site prediction of the GAS2 family members. Based on the PhosphoSitePlus online database (<http://www.phosphosite.org/homeAction.do>), the potential phosphorylation sites of GAS2 (A), GAS2L1 (B), GAS2L2 (C), and GAS2L3 (D) are predicted. Further, the reported phosphorylation sites, including the GAS2L1 T352 by Nek2A, and the GAS2L3 S307/S607 site by CDK1, have been marked.

### FIGURE S2. Mutation site analysis of the GAS2 family members in TCGA tumours. The specific mutation sites of GAS2 (A), GAS2L1 (B), GAS2L2 (C), and GAS2L3 (D) for the tumour samples of TCGA are analysed using the cBioPortal online website (<https://www.cbioportal.org/>).
